# Microbial Primer: Cooperation in bacteria

**DOI:** 10.1099/mic.0.001440

**Published:** 2024-04-01

**Authors:** Stuart A. West, Ashleigh S. Griffin

**Affiliations:** Department of Biology, University of Oxford, England, UK

**Keywords:** altruism, inclusive fitness, kin selection, cheat, public good

## Abstract

The growth and success of many bacteria appear to rely on a stunning range of cooperative behaviours. But what is cooperation and how is it studied?

## What Is Cooperation?

A trait or behaviour is defined as cooperation if it provides a benefit to another individual and is favoured by natural selection at least partially because of that benefit. Just being ‘helpful’ is not enough: the last clause is required to avoid traits that provide a benefit to others just as a by-product of an otherwise selfish behaviour.

For example, when an elephant produces dung, this benefits dung beetles that rely on dung for nutrition and somewhere to breed. But it obviously does not make sense to think of dung production as the result of elephants cooperating with beetles. The fact that beetles enjoy elephant dung does not tell us anything about the evolution of dung production in elephants.

This distinction might seem obvious in the dung scenario, but it can be a much more subtle in communities of bacteria. Two strains or species of bacteria may cross-feed on waste product produced by the other. That is just ‘eating each other’s poo’, not cooperation. Although, this could evolve into cooperation if a strain evolved to produce more of a waste product just to benefit the other strain (and hence obtain more benefit back).

## What Does Bacterial Cooperation Look Like?

The most common form of cooperation in bacteria is when cells produce and secrete factors that provide a benefit to the local group of cells. Examples include molecules to scavenge iron (siderophores), enzymes to break down proteins (proteases) and molecules to aid movement (rhamnolipids) This is analogous to the production of what economists call shared pool resources or ‘public goods’, and so they are commonly referred to as that, or as ‘extracellular factors’.

Public goods are not the only way that bacteria cooperate. Other forms include: (a) the production of fruiting bodies for dispersal; (b) utilizing a resource more efficiently and slowly; (c) a lower investment into competition for resources, such as lower growth rate; (d) apoptosis for the release of bacteriocins; (e) nitrogen fixing. As a shorthand, and to give a concrete case to think about, we shall focus the rest of this paper on public goods, but the same principles apply to any form of cooperation.

## How Do You Test If A Trait Is Cooperative?

**Fig. 1:**
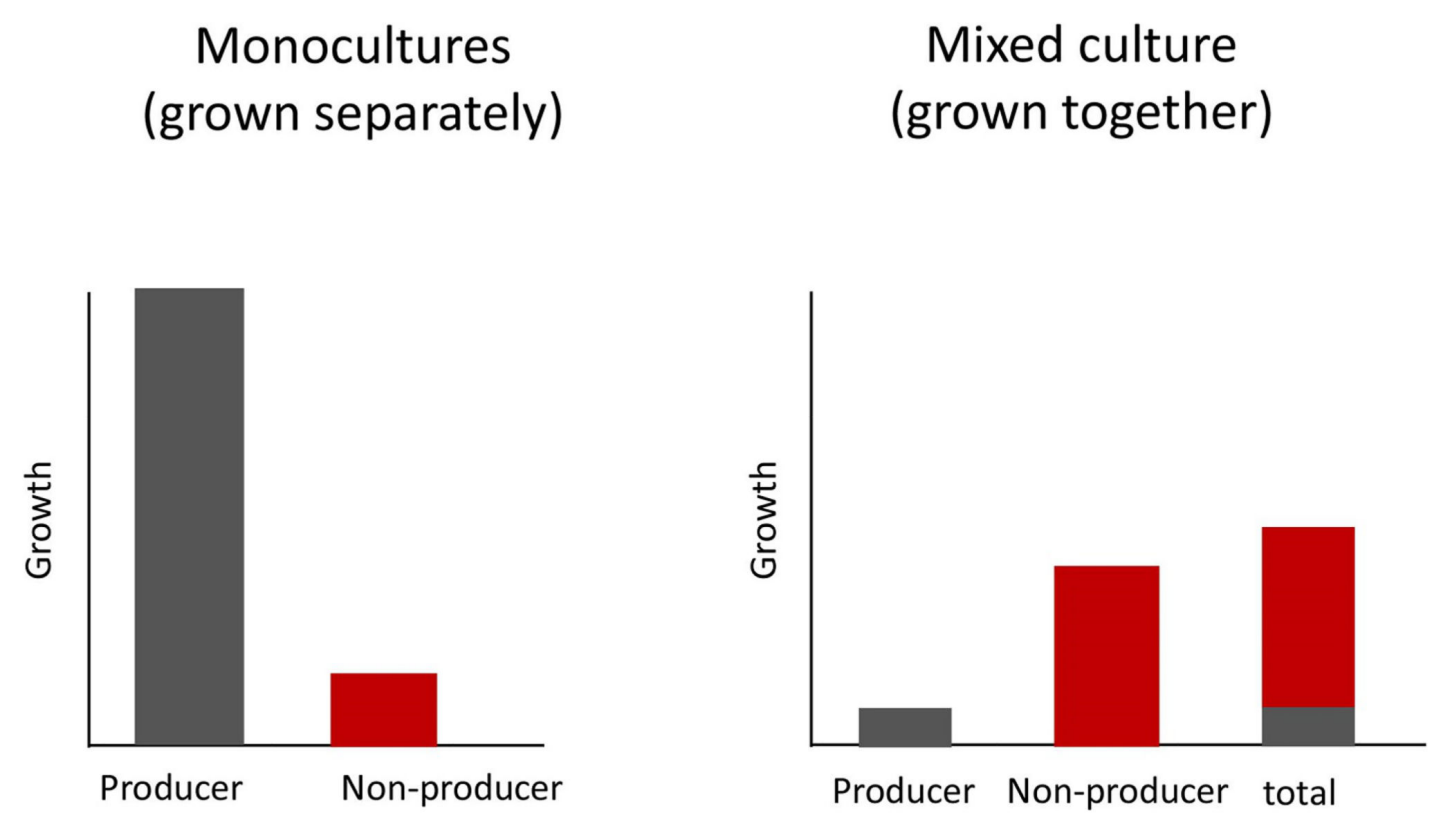
How to test for cooperation. We illustrate the hypothetical results of an experiment with a strain that produces a public good (putative cooperator) and a strain that does not produce the public good (putative cheat). These strains are grown separately (monoculture) and together (mixed culture). If the production of the public good is cooperative, then: (1) the producer has a higher growth rate (fitness) than the non-producer when each are grown separately; (2) the non-producer will have a higher growth than the producer when both are grown together. The overall total growth rate (both strains) when grown together will be lower than that of the producer when grown alone.

The fundamental first step is to examine the fitness consequences of the trait in a social context. This means examining the social costs and benefits by comparing the fitness of a strain (lineage) that produces a public good with that of a strain that does not produce the public good, when grown either separately or together (Fig. 1).

Let us consider a case where we have two strains that are genetically identical, except that one produces some public good and another does not. If this really is a cooperative public good, then we can make two predictions:

(1) When grown separately (monoculture) the group that produce the public good should grow better than the group that does not. This is because producing a public good (cooperation) provides a benefit to the local group of cells.
(2) When grown together (mixed culture), we predict the opposite pattern. The cells that do not produce the public good should outcompete the cells that do. This is because the benefit is shared between cells, but the cost is only paid by producers. Consequently, the non-producers can exploit (cheat) the cooperative production of the public good by others.

Such experiments have been carried out on a range of public goods, including siderophores, elastase and beta-lactamases in species such as *Pseudomonas aeruginosa, Bacillus subtilis, Stapylococcus aureus, Salmonella enterica* and *Myxococcus xanthus*. Analogous experiments have also been carried out for other forms of cooperation, and with other microbes, such as yeast and slime moulds. Once a cooperative effect has been detected, a whole slew of more detailed questions can be asked about the consequences of factors such as the environmental conditions, more subtle variation in the level of cooperation, population density or the relative frequency of producers and non-producers.

## In What Environments Should We Carry Out Such Experiments?

It is crucial to carry out these experiments in an environment where the trait is needed and that is as natural as possible. For example, if you examined the costs of benefits of siderophores in an environment where there was lots of freely available iron, and so siderophores are not needed or produced, then you would not be able to look at their cost and benefit. This would be like examining the cooperative nature of hunting in a zoo population of African wild dogs that were fed *ad lib* and did not hunt.

There have been increasing attempts to examine the costs and benefits of potentially cooperative traits in more natural environments. Early experiments were in liquid culture. Later experiments examined cultures growing on attached biofilms. Other experiments, on pathogenic species, examined growth during infections. In most cases, these confirmed the cooperative nature of producing public goods.

## Why Is Cooperation A Scientific Problem?

The potential problem with such cooperation is that it can be exploited by non-cooperators or ‘cheats’ that do no produce the public good but can still benefit from the public good produced by others. In the simplest possible case, cheats in a well-mixed population should initially increase in frequency and outcompete cooperators. Even though cooperation would provide a benefit in the long run at the species or group level, it does not mean that it will necessarily be favoured by natural selection. Explaining cooperation has been one of the greatest problems for evolutionary biology.

This problem of cooperation even occurs if cells gain some personal (direct) benefit from the production of a public good. What matters for natural selection is relative fitness – does the ability of a cheat to exploit cooperators allow them to increase in frequency? This can depend upon the extent to which public goods are shared between cells and the cost of producing the public good.

## So How Is Cooperation Maintained?

Given this problem, how is cooperation maintained? The simplest and probably most common solution is termed ‘kin selection’. Bacterial populations do not tend to be well-mixed. Clonal growth means that cells are highly likely to be growing next to and interacting with clone mates, to which they are genetically identical. This means that cooperation will be directed towards relatives who tend to share the gene for cooperation. When this is the case, cheats are less able to exploit cooperators and so cooperators will have higher fitness. The extreme case of this is analogous to when cheats and cooperators are grown in monoculture, and so cheats cannot exploit cooperators at all.

The more general evolutionary principle here is that natural selection is predicted to lead to organisms that maximize their inclusive fitness. Inclusive fitness captures how individuals can influence the transmission of their genes to future generations via either their own reproductive success (direct fitness) or the reproductive success of other individuals with which they share genes (indirect fitness). Kin selection is the process by which individuals gain indirect fitness benefits from helping relatives.

## Is There Experimental Evidence For Kin Selection?

Experimental evolution has demonstrated the role of kin selection in producing public goods. Several experiments have been carried out in which populations are initiated with a mixture of: (a) cooperators that produce a public good and (b) cheats that do not produce the public good. Relatedness is then manipulated such that some populations are maintained at high relatedness and some at low relatedness. Several methods can be used to alter population structure and hence relatedness, including the number of cells used to initiate subpopulations (tubes or wells or animal hosts) or the extent to which populations are mixed.

**Fig. 2:**
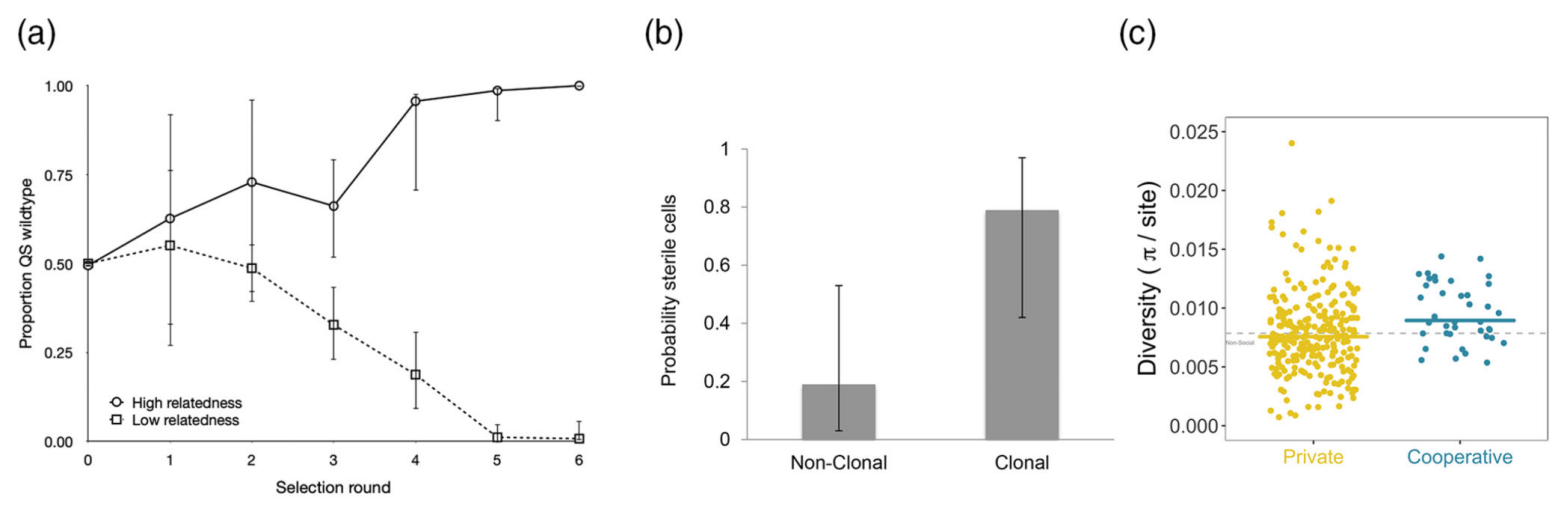
Kin selection for cooperation in bacteria. (a) Experimental evolution with *P. aeruginosa* infecting mice. A cooperative quorum-sensing strain (wild-type) outcompetes a non-cooperative (cheat) strain that does not quorum sense when at high relatedness, but the reverse happens at low relatedness. From 8. (b) Comparative study across species. Species that form groups clonally (*r*=1) are more likely to have sterile helper cells than species that form groups by aggregation (*r*≤1). From 6. (c) The population genetics of kin selection for cooperation. In *P. aeruginosa*, genes for cooperative traits show significantly higher diversity than genes for private traits. From 1.

A very repeatable pattern is that when populations are maintained at high relatedness the cooperators win and the cheats are eliminated (Fig. 2a). In contrast, when populations are maintained at low relatedness, the cheats win and the cooperators are eliminated. Consequently, cooperation is maintained by kin selection in populations at high but not low relatedness.

## But What About In The Real World?

Laboratory studies over the last 20 years or so have revolutionized our understanding of cooperation in bacteria. One challenge is to apply this to cooperation in ‘the wild’. Cooperation can be hard to study in natural populations of bacteria. If we were studying cooperation in an animal like a meerkat or a pied kingfisher, we could just watch them, and carry out simple manipulations, such as providing certain individuals with additional food. Unfortunately, it is very hard to watch bacteria in the wild. Luckily, there are at least two ways around this.

First, we can compare the biology of different species in across-species comparative studies. For example, some species, such as filamentous cyanobacteria, form clonal groups, where relatedness *r*=1. Other species form groups with the potential for some aggregation and so relatedness will often be lower (*r*≤1). Do we see higher levels of cooperation in the species where relatedness is higher? Yes, we do – they are more likely to have sterile helpers (Fig. 2b).

Second, we can look for evidence of kin selection at the genomic level. A genomic tool, SOCfinder, exists for finding genes for cooperative traits in bacteria. Population genetic theory suggests that kin selection will leave signatures (‘footprints’) at the genomic level, with genes for cooperative traits having increased polymorphism and divergence relative to genes for traits that provide personal (private) benefits to the cell. Studies on both *P. aeruginosa* and *B. subtilis* have found this pattern and suggest that the average relatedness between cells is approximately 0.8 (Fig. 2c). Comparisons can also be made across species. In the human microbiome, less common species, where relatedness is likely to be lower, have more genes for cooperative behaviours. Overall, the data from natural populations provide evidence for both cooperation and its maintenance by kin selection.

## Case Study: What Happens In Infections Of The Human Lung?

Long-term studies on *P. aeruginosa* infections in the lungs of patients with cystic fibrosis have allowed social dynamics to be studied over long time scales. The production of many public goods, such as siderophores, is gradually lost over time from strains infecting the human lung. This appears to be because they are outcompeted by cheats, who stop producing siderophores but maintain the ability to uptake them. The ability to uptake siderophores is only lost when all the other strains have also stopped producing them. The likely reason for the invasion of cheats is that the population structure within the human lung leads to a relatively low relatedness.

An alternative explanation for the loss of siderophore production in the human lung is that iron scavenging is no longer required. This alternative hypothesis can, however, be eliminated, because once siderophore production is lost, cells upregulate a different mechanism to obtain iron – *phu*. The *phu* system targets the iron-rich haem molecule, which is taken up directly through a membrane-bound receptor, PhuR. This mechanism appears to be less efficient than siderophores, but it is private and so cannot be exploited by cheats.

Consequently, what happens in the human lung is a three-step process: (1) cheats outcompete cooperators and so the production of a public good is lost; (2) individuals lose the ability to uptake the public good; (3) individuals upregulate an alternative and more costly private mechanism.

## Is Kin Selection The Only Way To Maintain Cooperation?

There can be other ways than kin selection to maintain cooperation, but these are relatively rare in bacteria. One possibility is that cooperation can be enforced by punishing non-cooperators or rewarding cooperators. Plants force bacteria to cooperate with them. Rhizobia fix nitrogen in the root nodules of legumous plants such as soybeans and provide it to the plants. Plants enforce this cooperative nitrogen fixation by cutting off the supply of resources to nodules where bacteria are not doing this. This form of enforcement has been called ‘sanctions’.

The term ‘group selection’ is sometimes suggested as a different mechanism to kin selection, but it is not. It is just a different way of doing the maths that leads to kin selection – and also predicts that inclusive fitness (not group fitness) is maximized. Most evolutionary biologists avoid group selection because it can be more limited, and often leads to confusion. Reinventions of kin selection are common, and other phrases that just mean kin selection include phenotypic similarity, assortment and network reciprocity.

## Should Cells Vary Their Level Of Cooperation?

The costs and benefits of cooperation can vary with environmental conditions, both biotic and abiotic. Consequently, if cells can assess this variation, they should vary their behaviour conditionally, and not just follow fixed (constitutive) strategies. For example, *P. aeruginosa* cells produce: (i) fewer iron scavenging siderophore molecules in iron-rich environments (when the benefit is lower) and (ii) more rhamnolipid biosurfactant where more carbon is available (when the cost is lower). This kind of conditional variation is sometimes called ‘phenotypic plasticity’.

## What About Quorum Sensing?

Quorum sensing appears to be a double form of cooperation! There is cooperation in both signalling and responding to the signalling. Quorum sensing is a process used to regulate several traits (behaviours) in many bacteria.

Bacteria produce and release small diffusible molecules, usually termed ‘signals’, which have two main consequences. First, the uptake of these molecules into cells regulates (autoinduction) a whole variety of behaviours, including the production of a range of public goods that are released from the cells to aid growth, motility, and/or biofilm formation.

Second, the uptake of these molecules also leads to an increase in the production of the signal molecule itself (autoregulation). The production of these signal or autoinducer molecules therefore leads to positive feedback at high cell densities, which results in a considerable increase in the production of signal- and quorum sensing-controlled public goods. The hypothesis here is that producing certain public goods is most beneficial at high cell densities, and that quorum sensing provides a mechanism that allows cells to increase the production of public goods at high cell density.

## Sounds Cool, But Is There Evidence For Quorum Sensing As Cooperatio?

There is evidence for quorum sensing as cooperation from both laboratory experiments and population genetics. Laboratory experiments with *P. aeruginosa* showed that both signalling and response to signalling are cooperative, in that they can be exploited by cheats that do not perform those behaviours. This same pattern has been observed when infecting hosts, with both *P. aeruginosa* (infecting mice) and *S. aureus* (infecting wax moth). In addition, laboratory experiments with *P. aeruginosa* have shown that the benefits of producing elastase, a public good controlled by quorum sensing, are greater at higher cell densities.

There is also evidence that quorum sensing is maintained by kin selection. Experimental evolution has shown that quorum sensing is favoured at high relatedness and lost at low relatedness in both *P. aeruginosa* and *S. aureus*. Population genetic analyses have shown signatures (footprints) of kin selection for cooperation in natural populations of both *P. aeruginosa* and *B. subtilis*. In both species, the public goods controlled by quorum sensing have increased polymorphism and divergence relative to private (intracellular) traits controlled by quorum sensing.

## Should We Expect Every Cell To Cooperate?

We should not expect every cell to cooperate, for at least two very different reasons. First, both cooperators and cheats can sometimes be maintained in the same population. This occurs when the relative fitness of cheats increases as they become rarer, because they are better able to exploit cooperators. This is termed ‘frequency dependence’ and allows cooperators and cheats to coexist.

Second, natural selection can sometimes favour variation between cooperating cells, where different cells specialize in different tasks. This is termed ‘division of labour’. For example, in *B. subtilis*, some cells produce proteases (a public good), while others do not. Other examples of division of labour include a fraction of *S. enterica* cells invading the gut tissue to trigger an inflammatory response that eliminates competing bacteria, and the stalk cells that hold up the viable spore cells in the fruiting bodies of *M. xanthus*. Differentiating cheating from division of labour is an important task when we see variation in a population.

## Can Cooperation Be Exploited?

The growth and virulence of many bacterial pathogens relies on cooperation. Indeed, when biologists talk about ‘virulence factors’, the production of which increases the virulence of a bacterial infection, they are pretty much always talking about a cooperative public good. This means that cooperation can be exploited to help manage pathogens.

The introduction of cheats into an infection can be used to reduce the mortality caused by infections. In *P. aeruginosa* the introduction of cheats was able to reduce the mortality rate in rats by 50%. This approach is also being trialled in viruses. Cheats can also be used as a ‘Trojan horse’ to introduce medically beneficial alleles into a population such as antibiotic suscetibility or antimicrobial toxins. Another possibility is that it can be harder for parasites to evolve resistance against treatments that disrupt cooperation than against treatments that directly kill them. This possibility has been supported in *Salmonella*, where resistance against standard antimicrobials rapidly evolves, but resistance to an inhibitor of cooperative biofilm formation did not evolve. Cheats can therefore offer novel ways to tackle pathogens in ways that are more ‘evolution-proof ’.

## Do We Really Need To Call It Cooperation?

We do not need to call things like siderophores cooperation, but there are huge advantages to doing so. Scientific progress depends upon precise, reliable communication between scientists. This can be hindered if people use different terms to mean the same thing, or the same term to mean different things. To give a deliberately extreme (and silly) example, imagine if some researchers started calling mitochondria ‘enzymes’ or used red and green interchangeably.

Another advantage of using cooperation is that bacteria are not the only organisms in which cooperation is studied. There is a much larger literature on cooperation across the tree of life that it is useful to link to. Hypotheses and ideas developed to explain or study cooperation in other organisms, such as animals, can be applied to help explain cooperation in bacteria. More generally, a major aim for evolutionary biology is to look for broad generalizations across species and taxa. Are there general patterns, that apply across different taxa, and make the world easier to explain? The importance of kin selection in explaining cooperation at all levels from bacteria to birds is one of the great success stories of evolutionary biology from this perspective.
